# Increase and Cause of Insanity

**Published:** 1907-12

**Authors:** 


### Increase and Cause of Insanity.

There are a number of known causes in the production of insanity, but heredity and alcoholism are the two most prominent factors. The ratio of insanity in Texas is increasing in proportion faster than the population. If the same ratio continues to exist the next ten years as it has in the past ten, we will need ten asylums to care for our insane, which will require the expenditure of three and one-half to four millions of dollars to maintain these institutions. In discussing the cause of insanity, Dr. T. C. Powell, who is today and has been superintendent of the Georgia Lunatic Asylum for over forty years, says: "Fifty per cent of all cases that have come under my observation, and perhaps a much larger per cent, is due to insane temperament. This inherited predisposition is due to the structural and physiological defects of the central nervous apparatus. But to what are these inherited tendencies due? They are more largely due to alcoholism than any other cause." He goes on further and says, "While a very small per cent of insanity is directly due to alcoholic liquors, from 50 to 75 per cent is indirectly due to it."

A large per cent of the most distinguished physicians of this country and Europe, who have given the subject of alcoholic heredity any investigation, say that from 50 to 75 per cent of insanity is caused directly by alcoholic liquors, visiting its effects upon the children by drunken parents.

Dr. Blanford, an eminent authority on insanity and its treatment, says: "Insanity increases in Europe and other countries in proportion to the amount of intoxicating liquors consumed."

Dr. Stearnes says: "From statistics it appears that all nervous diathesis, which is inherited, is due to alcohol, and there is no agent more disastrous and so potent in the production of brain disease and degeneration."

No well-informed man of today will deny the existence of alcoholic psychosis. To reject this doctrine is equivalent to denying the existence of God.

Alcoholic heredity and psychosis have been taught and recognized since the days of mythology, transmitting different forms of degeneracy, for which the present and future generations will pay heavy penalties.

At no other time since the dawn of civilization has the arrest of mental and physical development been so marked in your youth. Heart disease of children, alsmost unknown in the first years of the last century, are calling for methods of cure and institutions for their treatment. History supports the statement that in savage life, where we have no disturbing agencies, like alcohol, morphine, cocaine and tobacco, we have no insanity, but as we enter upon civilization and indulge in intoxicants, insanity develops and increases in proportion to the amount of alcohol consumed by the people. The direful effects of alcohol is not only ruining our present race, but the future generations by endowing their progeny with physical weakness and an everlasting thirst for alcohol. The etiological influence of alcohol on the human race is not understood by the masses. When its effects are taught and duly appreciated by the people, it will cease to play such an important part in the production of insanity. No other agent known to science will so completely bar success and impair health. By destroying and lowering the vitality of nerve structures, alcohol launches hereditary influences, and by the transmission gains momentum, and leaves its impact upon the nerve centers, and the mental faculties are demoralized, nerve energies hopelessly enfeebled. As a result of the foregoing, moral nature becomes lowered and the offspring endowed with moral and mental weakness, whose inevitable effect is early decay and death, because no agency competes with alcohol in lowering and depleting the powers of resistance to disease.

In support of the above views, I beg to quote Dr. Kraepelin, the most eminent authority on insanity and its treatment in this country, who says: "Over 33 per cent of insanity, epilepsy, idiocy and prostitution shows alcoholism in parents." Upon investigation as to the causes of the various mental disorders who daily come to this institution, we find that the relatives invariably say that the parents or grandparents were addicted to the use of intoxicants in a greater or less degree. This is ample proof that the baleful effects of alcohol are not altogether expended and confined to the generation addicted to it, but they are extended and invade generations to come.

In defending this contention, I submit the strongest evidence when God said he would visit the sins and iniquities of the father upon the children unto the third and fourth generation. When the people arouse to a sense of duty and appreciate the great danger attending the effects of alcohol, indulgence in the pernicious habit will be terminated. I find from careful examination that a large per cent of the young men who have been admitted to this institution of recent years have been addicted to cigarette smoking. I look upon the cigarette habit as being most baleful for young boys, and 95 per cent of the boys addicted to cigarette smoking will later use alcohol or its congeners, which often leads to stealing in order to secure means with which to purchase cigarettes and whisky. As the result of bad heredity and environments, twelve million boys and girls, or one-third of the children of the United States, are debarred from attending the schools of this country by reason of physical and mental weakness. We should not be surprised at this condition of affairs when the internal revenue receipts show that over two billion gallons of whisky, beer and wine were consumed in this country last year. Why be amazed at the increase of insanity, crime and degeneracy? This explains the question as to why the nation spends annually on the insane criminals and paupers "six billion dollars, while her annual wealth is only five billions." When the people of this country arrive at the point where they will obey the laws of God and nature and prohibit their children by law from intermarrying into families of weak diathesis, we will begin to improve physically, morally and mentally. Nerve impairment, however induced, is sure to reach the offspring. Hence the child of a drunkard is in the same category with the child of a lunatic.

It is a duty of those who are in a position to know these things to warn and teach the people the importance of building up the human race. More I will not say. I have shown that alcohol plays a great part in the rôle of insanity and that it is a most potent factor in the swift decay of the human race.—*From Annual Report of Dr. C. L. Gregory, Superintendent North Texas Hospital for Insane.*

